# Integrating Transcriptome-Wide Association Study and mRNA Expression Profiling Identifies Novel Genes Associated With Osteonecrosis of the Femoral Head

**DOI:** 10.3389/fgene.2021.663080

**Published:** 2021-06-07

**Authors:** Mei Ma, Peilin Li, Li Liu, Shiqiang Cheng, Bolun Cheng, Chu Jun Liang, Sijia Tan, Wenyu Li, Yan Wen, Xiong Guo, Cuiyan Wu

**Affiliations:** School of Public Health, Health Science Center, Xi’an Jiaotong University, Xi’an, China

**Keywords:** genome wide association study (GWAS), mRNA, disease-associated genes, osteonecrosis, transcriptome-wide association study (TWAS)

## Abstract

**Objective:**

This study aims to identify novel candidate genes associated with osteonecrosis of the femoral head (ONFH).

**Methods:**

A transcriptome-wide association study (TWAS) was performed by integrating the genome-wide association study dataset of osteonecrosis (ON) in the UK Biobank with pre-computed mRNA expression reference weights of muscle skeleton (MS) and blood. The ON-associated genes identified by TWAS were further subjected to gene ontology (GO) analysis by the DAVID tool. Finally, a trans-omics comparative analysis of TWAS and genome-wide mRNA expression profiling was conducted to identify the common genes and the GO terms shared by both DNA-level TWAS and mRNA-level expression profile for ONFH.

**Results:**

TWAS totally identified 564 genes that were with *P*_TWAS_ value <0.05 for MS and blood, such as *CBX1* (*P*_TWAS_ = 0.0001 for MS), *SRPK2* (*P*_TWAS_ = 0.0002 for blood), and *MYO5A* (*P*_TWAS_ = 0.0005 for blood). After comparing the genes detected by TWAS with the differentially expressed genes identified by mRNA expression profiling, we detected 59 overlapped genes, such as *STEAP4* [*P*_TWAS_ = 0.0270, FC (fold change)_mRNA_ = 7.03], *RABEP1* (*P*_TWAS_ = 0.010, FC_mRNA_ = 2.22), and *MORC3* (*P*_TWAS_ = 0.0053, FC_mRNA_ = 2.92). The GO analysis of TWAS-identified genes discovered 53 GO terms for ON. Further comparing the GO results of TWAS and mRNA expression profiling identified four overlapped GO terms, including cysteine-type endopeptidase activity (*P*_TWAS_ = 0.0006, *P*_mRNA_ = 0.0227), extracellular space (*P*_TWAS_ = 0.0342, *P*_mRNA_ = 0.0012), protein binding (*P*_TWAS_ = 0.0112, *P*_mRNA_ = 0.0106), and ATP binding (*P*_TWAS_ = 0.0464, *P*_mRNA_ = 0.0033).

**Conclusion:**

Several ONFH-associated genes and GO terms were identified by integrating TWAS and mRNA expression profiling. It provides novel clues to reveal the pathogenesis of ONFH.

## Introduction

Osteonecrosis (ON) is a common orthopedic disorder, the pathological features of which is the death of bone cells owing to the decrease of blood flow (Assouline-Dayan et al., 2002). Although ON can occur at different skeletal sites, such as the hips, jaw, knees, shoulders, and ankles, the femoral head is the mostly affected one. There are 20,000 to 30,000 new cases of osteonecrosis of the femoral head (ONFH) in the United States every year, of which about 5–10% end up with total hip replacement (Malizos et al., 2007).

Osteonecrosis is a complex multifactorial disease which is affected by both genetic elements and environmental factors (Baek et al., 2017). The risk factors for developing ON include serious trauma, corticosteroid medications, immunosuppressive therapy, autoimmune diseases, and chronic alcohol intake (Mont et al., 2000; Gladman et al., 2001). During the past decades, a number of genetic factors for ON have been conducted, linking specific genes or susceptibility loci to the pathogenesis of ON (Hadjigeorgiou et al., 2008; Karol et al., 2015; Sun et al., 2015; Zhou et al., 2015). For example, a meta-analysis study reported that vascular endothelial growth factors, endothelial nitric oxide synthase, and ATP-binding cassette subfamily B member 1 transporter (ABCB1) polymorphisms were associated with the risk of ONFH (Zhou et al., 2015). Another study observed that glucocorticoid-associated ON was associated with the genetic locus near the glutamate receptor gene (Karol et al., 2015). The R192Q and rs662 polymorphisms in paraoxonase-1 were also reported to increase the susceptibility of ONFH (Hadjigeorgiou et al., 2007; Li et al., 2017). Hypofibrinolysis conferred by the 4G/4G plasminogen activator inhibitor-1 gene variant is a major predisposing factor for avascular ON in renal transplant patients (Ferrari et al., 2002). The genetic polymorphisms in ABCB1 gene (C3435T), apolipo-protein B (ApoB) gene (C7623T), and cAMP-response element binding protein-binding protein (CBP) gene (rs3751845) increased and were helpful for predicting the risk of steroid-induced ONFH (Kuribayashi et al., 2008). However, previous studies mostly focused on single or several gene defects associated with ONFH, and a few large-scale genetic studies of ONFH have been conducted. The genetic mechanism of ON remains elusive now.

Genome-wide association studies (GWAS) is a powerful approach for identifying the susceptibility genes of complex diseases or traits. However, a great number of genetic variants affect complex traits by regulating gene expression and then changing the abundance of one or multiple proteins (Lappalainen et al., 2013; Zhang et al., 2015). For instance, the non-coding regulatory loci, such as expression quantitative trait loci and methylation quantitative traits loci (Grubert et al., 2015), can affect the risk of diseases through regulating the expression levels of disease-related genes. The GWAS-identified genetic loci are mostly located in the non-coding regulatory regions of genome. These causal genetic variants within non-coding regulatory regions are commonly indistinguishable from the neighboring markers and are likely to be missed in previous GWAS (Zhang and Lupski, 2015). In recent years, transcriptome-wide association study (TWAS) has been proposed, which is capable of identifying disease-associated genes at the mRNA expression level (Gusev et al., 2016a). TWAS has been applied to the genetic studies of multiple complex human diseases and presents good performance for disease gene mapping (Gusev et al., 2016b; Thériault et al., 2017). For instance, Gusev et al. (2016b) performed a TWAS of schizophrenia through integrating a GWAS dataset of schizophrenia and mRNA expression references from the brain, blood, and adipose tissues. Finally, they identified 157 schizophrenia-associated genes, of which 35 were novel. Wu et al. (2018) performed a TWAS to evaluate the associations between genetically predicted gene expression level and breast cancer risk and identified 48 candidate genes for breast cancer.

In this study, using the latest GWAS dataset of ON obtained from the UK Biobank, we first conducted a TWAS to scan candidate genes for ON. The ON-associated genes identified by TWAS were further subjected to gene ontology (GO) enrichment analysis by DAVID tool. To validate the TWAS results of ON, we also compared the TWAS results with the mRNA expression profiles of ONFH to identify common genes and GO terms shared by TWAS and mRNA expression profiling.

## Materials and Methods

### GWAS Summary Dataset of ON

The GWAS summary dataset of ON was driven from the UK Biobank database^1^ (Bycroft et al., 2018; Canela-Xandri et al., 2018). Briefly, the UK Biobank genetic dataset contains genome-wide genotype data for 452,264 participants, including 603 osteonecrosis patients, as defined by the International Classification of Diseases, Tenth Revision, (ICD-10) code “M87.” DNA was extracted from frozen-stored blood samples and performed for genotyping using the marker content of the UK Biobank Axiom array. The samples were imputed by a new version of the program referred to as IMPUTE4.^2^ Principal component analysis was applied to account for the population structure in both sample and marker-based quality control. The GWAS summary data contain 623,94 genotyped variants that passed quality control, 9,113,133 imputed variants that passed quality control, all 30,798,054 imputed variants available for downloading, and 9,113,133 imputed variants that passed quality control with a P different than 0 [for detailed information of the subjects, genotyping, imputation, and quality control, refer to the published studies (Bycroft et al., 2018; Canela-Xandri et al., 2018)].

### Gene Expression Profile of BMSCs

The mRNA expression profiling data of bone marrow mesenchymal stem cells (BMSCs) of ONFH patients was used here (Wang et al., 2018). Briefly, three patients with steroid-induced ONFH and three control subjects were enrolled from the Department of Orthopedic. ONFH was diagnosed based on preoperative radiographs and magnetic resonance images. Arraystar Human lncRNA microarray V3 (GPL16956), covering 26,109 mRNAs, was used for microarray analysis. Unpaired Student’s *t*-test was performed to evaluate the differences between the two groups. False discovery rate controlling was used to correct the *P*-value with Benjamini–Hochberg algorithm in R 3.4.1 suite (Lucent Technologies). Differentially expressed mRNAs were identified at | fold change (FC)| > 2.0 and Benjamini–Hochberg-corrected *P* values < 0.05. A total of 838 up-regulated mRNAs and 1,937 down-regulated mRNAs were identified in the ONFH group [for a detailed description of samples, experimental design, statistical analysis, and quality control, refer to the previous study (Wang et al., 2018)].

### TWAS of ON

TWAS of ON was performed using the FUSION software^3^ through integrating the UK Biobank ON GWAS summary data and pre-computed gene expression reference weights of peripheral blood, whole blood, and muscle skeleton (Gusev and Ko, 2016). Briefly, the gene expression weights of a certain tissue were first calculated using the prediction models implemented in FUSION. For a given gene, Bayesian sparse linear mixed model (Zhou et al., 2013b) was firstly used to compute SNP expression weights in the 1-Mb cis locus: let *w* denote weights, *Z* denote ON *Z* scores, and *L* denotes SNP correlation (LD) matrix. The formula “*Z*_TWAS_ = *w*′*Z*/(*w*′Lw)^1/2^” was then used to estimate the association between predicted expression and ON (Gusev and Ko, 2016). Finally, we got the gene–disease association by performing the expression imputation on chromosome one by one. In this study, the gene expression reference weight panels of peripheral blood (*n* = 1,247), whole blood (*n* = 1,264), and muscle skeleton (*n* = 361) were downloaded from the FUSION website.^4^ *P* value was calculated by FUSION for each gene.

### Gene Ontology Enrichment Analysis

The ON-associated genes identified by TWAS were further analyzed by the Database for Annotation, Visualization, and Integrated Discovery (DAVID)^5^ for GO enrichment (Huang da et al., 2009). The differently expressed mRNA of ONFH was also subjected to GO enrichment analysis. Finally, the GO enrichment results of TWAS and mRNA expression profile were compared to identify common GO terms for ONFH.

### Ethics

Our research data was downloaded from an online public database and does not involve ethical issues.

## Results

### TWAS Results of ON

Transcriptome-wide association study of ON identified 154 genes with *P* value < 0.05 in MS, such as STPG1 (*P*_TWAS_ = 0.0015), CTSS (*P*_TWAS_ = 0.0022), and THEM4 (*P*_TWAS_ = 0.01). The total 154 significant genes were presented in Supplementary Table 1. We also identified 128 genes with *P* values < 0.05 in peripheral blood (Supplementary Table 2) and 279 genes with *P* values < 0.05 in whole blood (Supplementary Table 3), such as GLT25D2 (*P*_TWAS_ = 0.0078), VAMP4 (*P*_TWAS_ = 0.0080), USP24 (*P*_TWAS_ = 0.0022), and LAPTM5 (*P*_TWAS_ = 0.0027). The top 10 significant genes identified by TWAS for ON are shown in Table 1.

**TABLE 1:** Top 10 genes identified by transcriptome-wide association study (TWAS) for osteonecrosis.

Tissue	Gene	Full gene name	TWAS.Z	Chr	*P* value
Muscle skeleton	CBX1	Chromobox 1	3.8600	17	0.0001
	DHODH	Dihydroorotate dehydrogenase	–3.2597	16	0.0011
Blood	SRPK2	SRSF protein kinase 2	–3.7146	7	0.0002
	CHMP1A	Charged multivesicular body protein 1A	3.6124	16	0.0003
	HP	Haptoglobin	3.4296	16	0.0006
	MYO5A	Myosin VA	–3.4933	15	0.0005
	KIAA1967 (CCAR2)	Cell cycle and apoptosis regulator	–3.3711	8	0.0007
	CRLF3	Cytokine receptor like factor 3	–2.6398	17	0.0083
	FAM113A (PCED1A)	PC-esterase domain containing 1A	3.2609	20	0.0011
	ANKS1A	Ankyrin repeat and sterile alpha motif domain containing 1A	–3.2490	6	0.0012

*Chr, chromosome; TWAS.Z, TWAS Z-score.*

### Common Genes Shared by TWAS and mRNA Expression Profiling

We further compared the genes identified by TWAS of ON with the differently expressed genes identified by mRNA expression profiling of ONFH. We detected 59 common genes shared by TWAS and mRNA expression profiling (Supplementary Table 4), such as RGPD8 (*P*_TWAS_ = 0.0060, FC_mRNA_ = 2.97), RABEP1 (*P*_TWAS_ = 0.0099, FC_mRNA_ = 2.22), STEAP4 (*P*_TWAS_ = 0.0273, FC_mRNA_ = 7.03), KDM5A (*P*_TWAS_ = 0.0202, FC_mRNA_ = 2.17), KLHL24 (*P*_TWAS_ = 0.0347, FC_mRNA_ = 2.36), and PDLIM1 (*P*_TWAS_ = 0.0473, FC_mRNA_ = 2.14). The top 10 common genes shared by TWAS and mRNA expression profiling are presented in Table 2.

**TABLE 2:** Common genes detected by both transcriptome-wide association study (TWAS) and mRNA expression profiles of bone marrow mesenchymal stem cells.

Gene	Full gene name	TWAS.Z	Chr	*P* value	FC	Regulation
HP	Haptoglobin	3.4296	16	0.0006	2.05	Up
PLA2G12A	Phospholipase A2 group XIIA	–2.8763	4	0.0040	2.06	Up
USP24	Ubiquitin-specific peptidase 24	3.0655	1	0.0022	2.72	Down
ANAPC4	Anaphase-promoting complex subunit 4	–2.9839	4	0.0027	2.02	Down
GKAP1	G kinase anchoring protein 1	–2.8968	9	0.0038	2.01	Down
MORC3	MORC family CW-type zinc finger 3	2.7881	21	0.0053	2.92	Down
RGPD8	RANBP2 like and GRIP domain containing 8	2.7454	2	0.0060	2.97	Down
CCDC125	Coiled-coil domain containing 125	2.6381	5	0.0083	2.32	Down
TDRD6	Tudor domain containing 6	2.6360	6	0.0084	13.45	Down
RABEP1	Rabaptin, RAB GTPase binding effector protein 1	2.5776	17	0.0099	2.22	Down

*Chr, chromosome; TWAS.Z, TWAS Z-score; FC, fold change.*

### GO Enrichment Analysis Results

Gene ontology enrichment analysis of the genes identified by TWAS detected 53 GO terms with *P* value < 0.05 for ON, such as mitochondrial matrix (*P* value = 0.0027), RNA catabolic process (*P* value = 2.91 × 10^–4^), and membrane (*P* value = 0.0096). Further comparing the GO enrichment analysis results of TWAS and mRNA expression profiling detected four common GO terms, including cysteine-type endopeptidase activity (*P*_TWAS_ = 0.0006, *P*_mRNA_ = 0.0227), extracellular space (*P*_TWAS_ = 0.0342, *P*_mRNA_ = 0.0012), protein binding (*P*_TWAS_ = 0.0112, *P*_mRNA_ = 0.0106), and ATP binding (*P*_TWAS_ = 0.0464, *P*_mRNA_ = 0.0033).

## Discussion

Limited efforts have been paid to explore the genetic mechanism of ONFH by now. The genes implicated in the development of ON remain largely unknown. In this study, we conducted a genome-wide integrative analysis of TWAS and mRNA expression profiling by identifying multiple ONFH-associated genes, such as STEAP4, RABEP1, and MORC3.

STEAP4 encodes a protein that belongs to the six transmembrane epithelial antigens of prostate (STEAP) family and resides in the Golgi apparatus. Previous studies demonstrated that STEAP4 is involved in responses to inflammatory and glucose metabolism (Wellen et al., 2007; ten Freyhaus et al., 2012; Kim et al., 2015). In addition, there is a study which identified that STEAP4 links to inflammation and colon cancer as a critical regulator of mitochondrial dysfunction (Xue et al., 2017). There has been no study report about STEAP4 in ON. However, one study indicated that STEAP4 had a critical role in cellular iron uptake and utilization in osteoclasts and was indispensable for osteoclast development and function (Zhou et al., 2013a). Impaired blood supply to the bone is associated with ON. Therefore, our result suggested that STEAP4 may be a regulator for iron uptake and utilization to link blood circulation and ON.

MORC3 encodes MORC family CW-type zinc finger protein 3, which localizes to the nuclear matrix. A previous study conducted by Jadhav et al. (2016) showed that MORC3 mutant mice exhibit bone cell differentiation. Furthermore, the localization of morc3 protein in (MUT±) osteoclasts and (MUT±) mice in the nuclear membrane to the cytoplasm, the localization of MORC3 protein in MORC3 (mut±) osteoclasts, and MORC3 (mut±) mice transferring from the nuclear membrane to the cytoplasm displayed increased osteoblast differentiation and altered gene expression (Jadhav et al., 2016). Another study demonstrated that MORC3 mutant mice exhibited reduced cortical thickness and area, followed by changed hematopoietic stem cell niche and bone cell differentiation (Hong et al., 2017).

RABEP1 encodes Rab GTPase-binding effector protein 1. It belongs to rabaptin protein family. It has been demonstrated that hypoxia was implicated in the development of bone diseases, including ON (Liu et al., 2015; Yin et al., 2020). A recent study investigated the role of hypoxia and hypoxia-inducible factor 1α (HIF-1α) in fibrodysplasia ossificans progressiva (FOP). They found that HIF-1α could increase the duration and intensity of BMP signaling through RABEP1-mediated retention of ACVR1 in hypoxic connective tissue progenitor cells from FOP patients (Wang et al., 2016). In addition, RABEP1 was identified as one of the novel candidate genes influencing spinal volumetric bone mineral density in rats (Alam et al., 2010).

Gene ontology enrichment analysis detected several GO terms, such as mitochondrial matrix, ATP binding, positive regulation of cell matrix adhesion, and RNA catabolic process. The mitochondrial matrix is the structural basis of energy metabolism and oxidative stress (Cadenas, 2018). A study indicated that steroid-associated mitochondrial injury and redox failure are important elements in the pathogenesis of ON (Tsuchiya et al., 2018). ATP binding is closely related to mitochondrial matrix. ABCB1 polymorphism contributes to the risk of ONFH (Zhou et al., 2015; Zhang et al., 2017). The positive regulation of cell matrix adhesion was identified to be implicated in the regulation of hypoxia (Zhang et al., 2018), which is one of the factors that cause apoptosis of bone cells (Seamon et al., 2012). In addition, RNA catabolic process is another identified GO enrichment term. Previous studies have demonstrated that RNA stability provides a rapid level of regulation that can have major effects in maintaining global inflammation (Herman and Autieri, 2018; Nyati et al., 2020).

To the best of our knowledge, this is the first TWAS of ON and identified multiple candidate genes whose imputed mRNA expression levels were associated with ON. To enhance the reliability and persuasiveness of our study, we further compared the TWAS results with the mRNA expression profiling of ONFH. We identified multiple common genes and GO terms shared by both DNA-level TWAS and mRNA expression profiling for ON. Despite that the power of TWAS is great, there are also two limitations that should be noted. Firstly, although TWAS is not confounded by reverse causality (disease→ expression independent of SNP), it is well nigh impossible to draw the instances of pleiotropy in statistics (where a SNP or linked SNPs affect ON and expression independently) and truly causal susceptibility genes. Secondly, there is some heterogeneity between GWAS data and gene expression profile. In detail, the GWAS data originate from European ON participants defined by ICD codes, while the gene expression data originate steroid-induced ONFH of Chinese ancestry. There has been lack of data set from the same ancestry and samples. Therefore, one should be careful in applying our study results, and further studies are needed to confirm our findings.

In summary, we conducted a genome-wide integrative analysis of TWAS and mRNA expression profiling of ON. We identified multiple candidate genes and their biological terms associated with ON. Our results provide novel clues for clarifying the pathogenesis of ON.

## Data Availability Statement

The original contributions presented in the study are included in the article/Supplementary Material, further inquiries can be directed to the corresponding author.

## Ethics Statement

Ethical review and approval was not required for the study on human participants in accordance with the local legislation and institutional requirements. The patients/participants provided their written informed consent to participate in this study.

## Author Contributions

MM and PL carried out the rSNP analysis and drafted the manuscript. LL, SC, BC, CL, ST, WL, YW, XG, and CW participated in its design and helped to draft the manuscript. All authors read and approved the final manuscript.

## Conflict of Interest

The authors declare that the research was conducted in the absence of any commercial or financial relationships that could be construed as a potential conflict of interest.

## Footnotes

1 http://geneatlas.roslin.ed.ac.uk/

2 https://jmarchini.org/software/

3 http://gusevlab.org/projects/fusion/

4 http://gusevlab.org/projects/fusion/

5 https://david.ncifcrf.gov/
