# Supplementary material for: Integrating Transcriptome-Wide Association Study and mRNA Expression Profiling Identifies Novel Genes Associated With Osteonecrosis of the Femoral Head
Source: Front Genet. 2021 Jun 7;12:663080. doi: 10.3389/fgene.2021.663080 (PMC8215574; doi:10.3389/fgene.2021.663080)
Supplement: Supplementary file 1 [file Data_Sheet_1.docx]

**Table S1 TWAS results of osteonecrosis using Muscle Skeleton gene expression weights**

| **Gene Name** | **Chromosome** | **TWAS.Z** | **TWAS.P** |
| --- | --- | --- | --- |
| STPG1 | 1 | 3.17447 | 0.0015 |
| MAST2 | 1 | -3.12801 | 0.00176 |
| CTSS | 1 | -3.06492 | 0.00218 |
| THEM4 | 1 | 2.57709 | 0.00996 |
| BEND5 | 1 | -2.53412 | 0.01127 |
| NSUN4 | 1 | -2.50447 | 0.01226 |
| SGIP1 | 1 | 2.37322 | 0.01763 |
| TPRG1L | 1 | 2.33048 | 0.01978 |
| TCEANC2 | 1 | 2.32171 | 0.02025 |
| MED8 | 1 | -2.17 | 0.03001 |
| EMC1 | 1 | -2.14345 | 0.03208 |
| RP11-316M1.12 | 1 | 2.1314 | 0.03306 |
| RP1-92O14.6 | 1 | -2.11468 | 0.03446 |
| RP1-43E13.2 | 1 | -2.10902 | 0.03494 |
| FNDC5 | 1 | -2.08773 | 0.03682 |
| RP11-96L14.7 | 1 | 2.05253 | 0.04012 |
| ZNF593 | 1 | 2.03234 | 0.04212 |
| RP11-305E17.6 | 1 | -2.02449 | 0.04292 |
| CERS2 | 1 | 1.9913 | 0.04645 |
| GSTM4 | 1 | -1.96913 | 0.04894 |
| ALX3 | 1 | -1.96398 | 0.04953 |
| CPS1 | 2 | -2.7734 | 0.00555 |
| RGPD8 | 2 | 2.7454 | 0.00604 |
| CASP8 | 2 | 2.4417 | 0.01462 |
| CEP68 | 2 | 2.4059 | 0.01613 |
| ALS2CR12 | 2 | -2.2991 | 0.0215 |
| STK39 | 2 | -2.2913 | 0.02195 |
| FASTKD1 | 2 | -2.2049 | 0.02746 |
| MERTK | 2 | -2.1754 | 0.0296 |
| ICA1L | 2 | 2.0797 | 0.03756 |
| GLS | 2 | -2.023 | 0.04307 |
| DYNC1I2 | 2 | -1.9744 | 0.04834 |
| NEK11 | 3 | 2.9837 | 0.00285 |
| ASTE1 | 3 | -2.9557 | 0.00312 |
| TMEM110 | 3 | 2.7143 | 0.00664 |
| ITIH4-AS1 | 3 | 2.5562 | 0.01058 |
| RP11-141C7.4 | 3 | 2.5498 | 0.01078 |
| ABCF3 | 3 | -2.5412 | 0.01105 |
| LNP1 | 3 | 2.3045 | 0.0212 |
| ANAPC13 | 3 | 2.2356 | 0.02538 |
| RHOA | 3 | 2.2296 | 0.02577 |
| RP11-767C1.2 | 3 | 2.1821 | 0.0291 |
| CEP63 | 3 | 2.1287 | 0.03328 |
| SDHAP2 | 3 | 2.1238 | 0.03369 |
| RP5-966M1.6 | 3 | 2.1095 | 0.0349 |
| PCOLCE2 | 3 | 2.0207 | 0.04331 |
| RP11-755B10.4 | 3 | 1.9711 | 0.04871 |
| AGA | 4 | 3.1814 | 0.00147 |
| ANAPC4 | 4 | -2.9989 | 0.00271 |
| PI4K2B | 4 | -2.9861 | 0.00283 |
| ZCCHC4 | 4 | -2.6305 | 0.00853 |
| Z95704.2 | 4 | -2.3255 | 0.02004 |
| MED28 | 4 | 2.0982 | 0.03588 |
| RP11-472B18.1 | 4 | -2.0305 | 0.0423 |
| MRPS18C | 4 | -1.9869 | 0.04694 |
| RN7SL689P | 5 | 3.11091 | 0.00187 |
| RP11-359P5.1 | 5 | 2.65844 | 0.00785 |
| CCDC125 | 5 | 2.63811 | 0.00834 |
| PPIC | 5 | 2.60704 | 0.00913 |
| CDK7 | 5 | -2.35889 | 0.01833 |
| SLC36A1 | 5 | 2.26802 | 0.02333 |
| 6-Mar | 5 | -2.02001 | 0.04338 |
| WASF1 | 6 | 2.8647 | 0.00417 |
| MDGA1 | 6 | -2.7279 | 0.00637 |
| TDRD6 | 6 | 2.636 | 0.00839 |
| SLC25A27 | 6 | -2.0717 | 0.03829 |
| MLLT4 | 6 | -1.9878 | 0.04683 |
| CCT6P1 | 7 | -2.35334 | 0.0186 |
| RP11-458F8.4 | 7 | 2.3402 | 0.0193 |
| KBTBD2 | 7 | 2.326 | 0.02 |
| STAG3L5P-PVRIG2P-PILRB | 7 | 2.17349 | 0.0297 |
| PILRA | 7 | 2.17415 | 0.0297 |
| ZCWPW1 | 7 | 2.14693 | 0.0318 |
| TSPAN33 | 7 | -2.11216 | 0.0347 |
| RSBN1L-AS1 | 7 | 2.10542 | 0.0353 |
| TSC22D4 | 7 | 2.06035 | 0.0394 |
| INTS4L1 | 7 | -1.99286 | 0.0463 |
| DOCK5 | 8 | -2.9243 | 0.00345 |
| WDYHV1 | 8 | 2.5733 | 0.01007 |
| CA8 | 8 | 2.3057 | 0.02112 |
| CCAR2 | 8 | -2.1838 | 0.02897 |
| CTD-2336O2.1 | 8 | 2.0589 | 0.0395 |
| C8orf58 | 8 | -1.9626 | 0.0497 |
| RP11-509J21.1 | 9 | 2.4724 | 0.0134 |
| RP11-535M15.2 | 9 | -2.3052 | 0.0212 |
| GOLM1 | 9 | -2.1483 | 0.0317 |
| RP11-112J3.16 | 9 | -2.09 | 0.0366 |
| LIPA | 10 | 2.981 | 0.00287 |
| PPA1 | 10 | -2.61452 | 0.00894 |
| OBFC1 | 10 | -2.47865 | 0.01319 |
| ITIH5 | 10 | 2.20084 | 0.02775 |
| AKR1C2 | 10 | 2.12987 | 0.03318 |
| ZNF33B | 10 | -2.10031 | 0.0357 |
| CWF19L1 | 10 | -2.06488 | 0.03893 |
| C10orf11 | 10 | -2.01436 | 0.04397 |
| KIN | 10 | -1.99153 | 0.04642 |
| EIF3F | 11 | -2.79814 | 0.00514 |
| MED17 | 11 | 2.36064 | 0.01824 |
| TRIM66 | 11 | 2.27213 | 0.02308 |
| FDX1 | 11 | -2.17081 | 0.02995 |
| ALG8 | 11 | 2.16701 | 0.03023 |
| ARL2 | 11 | -2.10616 | 0.03519 |
| C11orf54 | 11 | 1.98489 | 0.04716 |
| GOLGA2B | 12 | -2.6394 | 0.00831 |
| ARL1 | 12 | 2.0398 | 0.04137 |
| NAV3 | 12 | -2.007 | 0.04475 |
| PARP4 | 13 | 3.1679 | 0.00154 |
| ACOT1 | 14 | -2.8557 | 0.00429 |
| EIF2S1 | 14 | 2.3339 | 0.0196 |
| GLRX5 | 14 | 2.1962 | 0.02808 |
| ACOT1 | 14 | -2.8557 | 0.00429 |
| EIF2S1 | 14 | 2.3339 | 0.0196 |
| GLRX5 | 14 | 2.1962 | 0.02808 |
| DHODH | 16 | -3.25973 | 0.00112 |
| AC005363.9 | 16 | -2.54399 | 0.01096 |
| COQ9 | 16 | -2.30702 | 0.02105 |
| GAS8 | 16 | -2.27921 | 0.02265 |
| CLUAP1 | 16 | -2.06271 | 0.03914 |
| URAHP | 16 | -1.99168 | 0.04641 |
| MPHOSPH6 | 16 | -1.969 | 0.04895 |
| CBX1 | 17 | 3.86 | 0.000113 |
| TEFM | 17 | -3.1308 | 0.001743 |
| SUZ12P | 17 | -2.654 | 0.007955 |
| PSMC5 | 17 | -2.6333 | 0.008456 |
| FBF1 | 17 | -2.6322 | 0.008484 |
| RABEP1 | 17 | 2.5776 | 0.009948 |
| AFMID | 17 | -2.576 | 0.009995 |
| AC003665.1 | 17 | -2.4947 | 0.012608 |
| SRR | 17 | 2.3404 | 0.019261 |
| MIEF2 | 17 | 2.326 | 0.020019 |
| ARHGAP44 | 17 | -2.0267 | 0.042696 |
| ZADH2 | 18 | -2.3384 | 0.0194 |
| TPGS2 | 18 | 2.233 | 0.0255 |
| PLD3 | 19 | 2.54326 | 0.011 |
| VRK3 | 19 | -2.5178 | 0.0118 |
| RTN2 | 19 | -2.37708 | 0.0175 |
| ZNF100 | 19 | -2.22634 | 0.026 |
| CTD-2583A14.8 | 19 | -2.22056 | 0.0264 |
| ZNF180 | 19 | 2.20185 | 0.0277 |
| FARSA | 19 | 2.12832 | 0.0333 |
| PLEKHF1 | 19 | 2.06516 | 0.0389 |
| ZNF587 | 19 | -1.99341 | 0.0462 |
| HSD17B14 | 19 | 1.96245 | 0.0497 |
| EIF6 | 20 | -2.844 | 0.00446 |
| RP4-614O4.11 | 20 | -2.7262 | 0.00641 |
| MMP24-AS1 | 20 | 2.5427 | 0.011 |
| FER1L4 | 20 | -2.4092 | 0.01599 |
| MMP24 | 20 | 2.0711 | 0.03835 |
| DNTTIP1 | 20 | 1.9862 | 0.04701 |
| VPS16 | 20 | -1.9793 | 0.04779 |
| PTTG1IP | 21 | 2.0848 | 0.0371 |
| COX5BP7 | 22 | -3.21164 | 0.00132 |
| CTA-217C2.1 | 22 | 2.81776 | 0.00484 |
| RP3-402G11.26 | 22 | -2.35923 | 0.01831 |

Note: TWAS, transcriptome-wide association study; TWAS.Z, TWAS Z-score; TWAS.P, TWAS *P*-value.

**Table S2 TWAS results of osteonecrosis using peripheral blood gene expression weights**

| **Gene Name** | **Chromosome** | **TWAS.Z** | **TWAS.P** |
| --- | --- | --- | --- |
| GLT25D2 | 1 | 2.661 | 0.00779 |
| VAMP4 | 1 | -2.651 | 0.00803 |
| PHACTR4 | 1 | -2.6315 | 0.0085 |
| USF1 | 1 | 2.5433 | 0.01098 |
| TOR1AIP1 | 1 | -2.5118 | 0.01201 |
| ZYG11B | 1 | -2.3821 | 0.01721 |
| LRRC7 | 1 | 2.326 | 0.02002 |
| NSUN4 | 1 | 2.3042 | 0.02121 |
| ANP32E | 1 | 2.2306 | 0.0257 |
| RPA2 | 1 | -2.1751 | 0.02962 |
| TPRG1L | 1 | -2.1341 | 0.03284 |
| RCSD1 | 1 | -2.1082 | 0.03501 |
| DISC1 | 1 | 2.0998 | 0.03575 |
| F11R | 1 | 2.0978 | 0.03592 |
| PAFAH2 | 1 | 2.082 | 0.03734 |
| C1orf100 | 1 | -2.0572 | 0.03966 |
| FUCA1 | 1 | 2.0355 | 0.0418 |
| TCEB3 | 1 | 2.0229 | 0.04308 |
| SNX27 | 1 | -2.0142 | 0.04399 |
| CTSK | 1 | -1.9951 | 0.04603 |
| S100A8 | 1 | -1.9856 | 0.04707 |
| MIER1 | 1 | -1.9755 | 0.04821 |
| ADD2 | 2 | 2.51259 | 0.012 |
| CD302 | 2 | -2.48515 | 0.0129 |
| CASP10 | 2 | 2.43795 | 0.0148 |
| OLA1 | 2 | -2.26991 | 0.0232 |
| CASP8 | 2 | 2.22487 | 0.0261 |
| UBE2F | 2 | 2.10134 | 0.0356 |
| MFSD6 | 2 | 2.02548 | 0.0428 |
| VAMP8 | 2 | 2.02392 | 0.043 |
| PAIP2B | 2 | 1.96241 | 0.0497 |
| WDR82 | 3 | 2.44723 | 0.0144 |
| ABCC5 | 3 | -2.38602 | 0.017 |
| ITIH4 | 3 | 2.33716 | 0.0194 |
| CEP63 | 3 | 2.32561 | 0.02 |
| ANAPC13 | 3 | 2.23874 | 0.0252 |
| ALPK1 | 4 | 2.8497 | 0.00438 |
| AGA | 4 | 2.81 | 0.00495 |
| MGST2 | 4 | 2.3898 | 0.01686 |
| CCDC109B | 4 | -2.3507 | 0.01874 |
| KLB | 4 | 2.3406 | 0.01925 |
| MED28 | 4 | 2.2142 | 0.02682 |
| CASP6 | 4 | -2.1699 | 0.03001 |
| QDPR | 4 | -2.0641 | 0.03901 |
| ZNF354A | 5 | 3.1578 | 0.00159 |
| CCL28 | 5 | 2.77357 | 0.00554 |
| GAPT | 5 | 2.54717 | 0.01086 |
| HNRNPAB | 5 | -2.27809 | 0.02272 |
| CCDC125 | 5 | 2.19946 | 0.02785 |
| FAM13B | 5 | 2.09592 | 0.03609 |
| CLTB | 5 | 1.9949 | 0.04605 |
| HMGXB3 | 5 | 1.99396 | 0.04616 |
| MDGA1 | 6 | -3.13725 | 0.00171 |
| RNASET2 | 6 | -3.03437 | 0.00241 |
| VNN3 | 6 | 2.783882 | 0.00537 |
| SLC22A23 | 6 | -2.0969 | 0.036 |
| PLN | 6 | -2.03949 | 0.0414 |
| DYNLT1 | 6 | -1.96307 | 0.04964 |
| CMAH | 6 | -1.96068 | 0.04992 |
| SRPK2 | 7 | -3.71456 | 0.000204 |
| C7orf25 | 7 | -2.70894 | 0.00675 |
| RSBN1L | 7 | 2.39135 | 0.016787 |
| FKBP9 | 7 | -2.27367 | 0.022986 |
| LRRC4 | 7 | 2.23493 | 0.025422 |
| STEAP4 | 7 | 2.20782 | 0.027257 |
| LRWD1 | 7 | 2.18579 | 0.028831 |
| PILRB | 7 | 2.17513 | 0.02962 |
| GUSB | 7 | -2.06242 | 0.039167 |
| TRBC2 | 7 | -2.0524 | 0.04013 |
| DLX6 | 7 | -2.04619 | 0.040737 |
| MEPCE | 7 | -2.02564 | 0.042802 |
| IGFBP3 | 7 | -1.96286 | 0.049663 |
| CPNE3 | 8 | -2.57521 | 0.01002 |
| PTK2B | 8 | -2.26656 | 0.02342 |
| GINS4 | 8 | 2.22846 | 0.02585 |
| R3HCC1 | 8 | -1.99251 | 0.04632 |
| STX17 | 9 | -2.2358 | 0.0254 |
| HIATL1 | 9 | -2.0287 | 0.0425 |
| tAKR | 10 | 2.58372 | 0.00977 |
| WDR37 | 10 | -2.34949 | 0.0188 |
| AKR1E2 | 10 | 2.29904 | 0.0215 |
| DHX32 | 10 | 2.18971 | 0.02855 |
| C10orf131 | 10 | 2.07019 | 0.03843 |
| CASP4 | 11 | -2.7336 | 0.00626 |
| TRIM66 | 11 | 2.4807 | 0.01311 |
| UCP2 | 11 | 2.179 | 0.02933 |
| KDM5A | 12 | -2.32181 | 0.0202 |
| TAOK3 | 12 | -2.27247 | 0.0231 |
| C12orf39 | 12 | -2.15152 | 0.0314 |
| DHRS12 | 13 | -2.1076 | 0.0351 |
| RNASE3 | 14 | 2.5503 | 0.0108 |
| HEATR4 | 14 | -2.1836 | 0.029 |
| RNASE2 | 14 | 2.0462 | 0.0407 |
| OSGEP | 14 | 1.991 | 0.0465 |
| C14orf105 | 14 | 1.9726 | 0.0485 |
| ISG20 | 15 | -2.56255 | 0.0104 |
| SYNM | 15 | 2.30046 | 0.0214 |
| SGK269 | 15 | -2.15307 | 0.0313 |
| NEO1 | 15 | -2.10138 | 0.0356 |
| HP | 16 | 3.4296 | 0.000604 |
| ZNF48 | 16 | -2.4593 | 0.013921 |
| POLR3K | 16 | 1.9896 | 0.046631 |
| NUP88 | 17 | 2.689 | 0.00717 |
| CRLF3 | 17 | -2.6398 | 0.00829 |
| MAP3K3 | 17 | 2.5756 | 0.01001 |
| SNX11 | 17 | 2.3463 | 0.01896 |
| LIMD2 | 17 | 2.3352 | 0.01953 |
| CDK5R1 | 17 | -2.3024 | 0.02131 |
| ACOX1 | 17 | 2.202 | 0.02766 |
| CPD | 17 | -2.0947 | 0.0362 |
| ACTG1 | 17 | -2.0821 | 0.03734 |
| PPP4R1 | 18 | 3.0715 | 0.00213 |
| NAPG | 18 | 2.3621 | 0.01817 |
| CEP192 | 18 | 2.3478 | 0.01888 |
| LMAN1 | 18 | -2.3066 | 0.02108 |
| ZADH2 | 18 | -2.1525 | 0.03135 |
| DSC1 | 18 | -2.103 | 0.03547 |
| MRPL54 | 19 | 2.41881 | 0.0156 |
| VRK3 | 19 | -2.39295 | 0.0167 |
| KLK1 | 19 | -2.31947 | 0.0204 |
| ZNF568 | 19 | 2.16744 | 0.0302 |
| ZNF66 | 19 | -2.04978 | 0.0404 |
| ZNF266 | 19 | -1.96598 | 0.0493 |
| FAM113A | 20 | 3.2609 | 0.00111 |
| CPNE1 | 20 | -2.1447 | 0.03198 |
| CD40 | 20 | -2.0137 | 0.04404 |
| ITSN1 | 21 | -2.2605 | 0.0238 |
| PIM3 | 22 | 2.006 | 0.0449 |

Note: TWAS, transcriptome-wide association study; TWAS.Z, TWAS Z-score; TWAS.P, TWAS *P*-value.

**Table S3 TWAS results of osteonecrosis using whole blood gene expression weights**

| **Gene Name** | **Chromosome** | **TWAS.Z** | **TWAS.P** |
| --- | --- | --- | --- |
| USP24 | 1 | 3.0655 | 0.00217 |
| LAPTM5 | 1 | -3.0018 | 0.00268 |
| TRNAU1AP | 1 | 2.9589 | 0.00309 |
| PHACTR4 | 1 | -2.939 | 0.00329 |
| S100A12 | 1 | -2.9092 | 0.00362 |
| PRPF3 | 1 | 2.9013 | 0.00372 |
| YTHDF2 | 1 | -2.805 | 0.00503 |
| ENO1 | 1 | -2.7653 | 0.00569 |
| RCC1 | 1 | -2.672 | 0.00754 |
| NSUN4 | 1 | 2.6579 | 0.00786 |
| THEM4 | 1 | 2.6014 | 0.00928 |
| TRIT1 | 1 | 2.4788 | 0.01318 |
| JMJD4 | 1 | 2.4444 | 0.01451 |
| TSEN15 | 1 | -2.4343 | 0.01492 |
| RER1 | 1 | 2.4209 | 0.01548 |
| RABGGTB | 1 | -2.3897 | 0.01686 |
| DCLRE1B | 1 | 2.3281 | 0.01991 |
| MCL1 | 1 | -2.3214 | 0.02027 |
| SCNM1 | 1 | 2.3157 | 0.02058 |
| FAAH | 1 | 2.2851 | 0.02231 |
| RCSD1 | 1 | -2.2778 | 0.02274 |
| RPRD2 | 1 | 2.2605 | 0.02379 |
| IPP | 1 | 2.2313 | 0.02566 |
| TCEB3 | 1 | 2.1706 | 0.02996 |
| CEP350 | 1 | 2.1329 | 0.03293 |
| PAFAH2 | 1 | 2.1149 | 0.03444 |
| FUCA1 | 1 | 2.0561 | 0.03978 |
| ARNT | 1 | -2.0248 | 0.04288 |
| MTHFR | 1 | 2.0135 | 0.04406 |
| MDM4 | 1 | 1.9986 | 0.04565 |
| PDZK1IP1 | 1 | 1.9606 | 0.04993 |
| MERTK | 2 | -2.82222 | 0.00477 |
| RMND5A | 2 | 2.6094 | 0.00907 |
| STRADB | 2 | 2.60063 | 0.00931 |
| ADAM23 | 2 | -2.56025 | 0.01046 |
| PPP1R7 | 2 | 2.43541 | 0.01487 |
| UNC50 | 2 | -2.32347 | 0.02015 |
| MDH1 | 2 | -2.31693 | 0.02051 |
| DDX1 | 2 | 2.31395 | 0.02067 |
| HAT1 | 2 | -2.30097 | 0.02139 |
| MAT2A | 2 | 2.24891 | 0.02452 |
| CDC42EP3 | 2 | 2.24493 | 0.02477 |
| LANCL1 | 2 | -2.2279 | 0.02589 |
| MFSD6 | 2 | 2.22289 | 0.02622 |
| GCA | 2 | -2.17361 | 0.02973 |
| FASTKD1 | 2 | -2.10518 | 0.03528 |
| SLC25A12 | 2 | 2.0593 | 0.03947 |
| VAMP8 | 2 | 2.03899 | 0.04145 |
| CD302 | 2 | -2.02777 | 0.04258 |
| MEIS1 | 2 | -2.02465 | 0.0429 |
| RQCD1 | 2 | -1.97732 | 0.04801 |
| SPCS1 | 3 | 3.1224 | 0.00179 |
| GLYCTK | 3 | -3.112 | 0.00186 |
| PPM1M | 3 | -2.7861 | 0.00534 |
| NT5DC2 | 3 | -2.6689 | 0.00761 |
| GNL3 | 3 | -2.6517 | 0.00801 |
| LPP | 3 | -2.5949 | 0.00946 |
| ATP2C1 | 3 | -2.5936 | 0.0095 |
| DAG1 | 3 | 2.5615 | 0.01042 |
| B4GALT4 | 3 | -2.4461 | 0.01444 |
| COPB2 | 3 | -2.421 | 0.01548 |
| WDR48 | 3 | -2.3258 | 0.02003 |
| NEK11 | 3 | 2.2597 | 0.02384 |
| PCNP | 3 | 2.2454 | 0.02475 |
| UBE2E2 | 3 | -2.2336 | 0.02551 |
| CEP63 | 3 | 2.2065 | 0.02735 |
| ANAPC13 | 3 | 2.1535 | 0.03128 |
| ITIH4 | 3 | 2.118 | 0.03418 |
| KLHL24 | 3 | 2.1122 | 0.03467 |
| CLEC3B | 3 | 2.0901 | 0.03661 |
| RAB7A | 3 | -2.0027 | 0.04521 |
| ACAA1 | 3 | -1.9777 | 0.04796 |
| MKRN2 | 3 | 1.9749 | 0.04828 |
| SRP72 | 4 | -3.05574 | 0.00225 |
| ANAPC4 | 4 | -2.98391 | 0.00285 |
| PLA2G12A | 4 | -2.87625 | 0.00402 |
| CAMK2D | 4 | -2.40658 | 0.0161 |
| CCDC109B | 4 | -2.25529 | 0.02411 |
| DCAF16 | 4 | 2.17 | 0.03001 |
| RNF4 | 4 | 2.14528 | 0.03193 |
| MFSD7 | 4 | -2.11086 | 0.03478 |
| 11-Sep | 4 | 2.09009 | 0.03661 |
| TET2 | 4 | 2.0142 | 0.04399 |
| AGA | 4 | 1.97109 | 0.04871 |
| ATG12 | 5 | 2.74287 | 0.00609 |
| AMACR | 5 | 2.73984 | 0.00615 |
| ITK | 5 | 2.44165 | 0.01462 |
| GAPT | 5 | 2.37568 | 0.01752 |
| ALDH7A1 | 5 | -2.31796 | 0.02045 |
| CLK4 | 5 | 2.22765 | 0.0259 |
| CCNH | 5 | -2.02353 | 0.04302 |
| SKP1 | 5 | -2.00268 | 0.04521 |
| ANKS1A | 6 | -3.24902 | 0.00116 |
| RNASET2 | 6 | -3.0956 | 0.00196 |
| MAP7 | 6 | -3.06082 | 0.00221 |
| RNF144B | 6 | -2.95176 | 0.00316 |
| STX7 | 6 | 2.91912 | 0.00351 |
| PREP | 6 | 2.51923 | 0.01176 |
| VNN2 | 6 | 2.46228 | 0.01381 |
| TRIM38 | 6 | 2.45563 | 0.01406 |
| MDGA1 | 6 | -2.42938 | 0.01512 |
| RWDD2A | 6 | 2.40776 | 0.01605 |
| VNN3 | 6 | 2.39718 | 0.01652 |
| ZNF76 | 6 | 2.20276 | 0.02761 |
| EEF1E1 | 6 | -2.18331 | 0.02901 |
| MYLK4 | 6 | 2.162 | 0.03062 |
| SMAP1 | 6 | 2.12871 | 0.03328 |
| PHF10 | 6 | -2.0682 | 0.03862 |
| CD83 | 6 | -2.0624 | 0.03917 |
| PEX7 | 6 | 2.05323 | 0.04005 |
| ENPP4 | 6 | 2.02009 | 0.04337 |
| LRWD1 | 7 | 2.74E+00 | 0.00614 |
| CLDN15 | 7 | 2.72E+00 | 0.00647 |
| SRI | 7 | 2.54E+00 | 0.01098 |
| FIGNL1 | 7 | 2.40E+00 | 0.0162 |
| ZNF777 | 7 | 2.29E+00 | 0.0219 |
| RAMP3 | 7 | 2.26E+00 | 0.02358 |
| PILRB | 7 | 2.22E+00 | 0.02618 |
| STEAP4 | 7 | 2.15E+00 | 0.03154 |
| ATP5J2 | 7 | 2.11E+00 | 0.0352 |
| TSC22D4 | 7 | 2.05E+00 | 0.04008 |
| DAGLB | 7 | 2.05E+00 | 0.04009 |
| HIP1 | 7 | 2.00E+00 | 0.04543 |
| WIPI2 | 7 | 1.96E+00 | 0.04972 |
| KIAA1967 | 8 | -3.3711 | 0.000749 |
| HSF1 | 8 | -2.6994 | 0.006947 |
| PTK2B | 8 | -2.4575 | 0.01399 |
| ASH2L | 8 | -2.3554 | 0.018501 |
| KCTD9 | 8 | 2.1747 | 0.029655 |
| CPNE3 | 8 | -2.08 | 0.037529 |
| FANCG | 9 | 3.126 | 0.00177 |
| GKAP1 | 9 | -2.8968 | 0.00377 |
| NOXA1 | 9 | -2.332 | 0.0197 |
| TRPM6 | 9 | 2.3051 | 0.02116 |
| AGTPBP1 | 9 | -2.1507 | 0.0315 |
| PPAPDC2 | 9 | -2.0828 | 0.03727 |
| ERP44 | 9 | -2.0602 | 0.03938 |
| ERMP1 | 9 | 2.0509 | 0.04027 |
| RAD23B | 9 | 2.0298 | 0.04238 |
| TEX10 | 9 | -2.0049 | 0.04497 |
| ALDH1A1 | 9 | -1.9619 | 0.04978 |
| PPA1 | 10 | -2.7441 | 0.00607 |
| DUSP5 | 10 | 2.5842 | 0.00976 |
| COMTD1 | 10 | 2.3681 | 0.01788 |
| NSUN6 | 10 | 2.2988 | 0.02152 |
| SMNDC1 | 10 | 2.2368 | 0.0253 |
| PDLIM1 | 10 | -1.9833 | 0.04733 |
| DENND5A | 11 | 2.893639 | 0.00381 |
| ZDHHC5 | 11 | -2.71176 | 0.00669 |
| FDX1 | 11 | -2.65489 | 0.00793 |
| PPP1R14B | 11 | 2.639449 | 0.0083 |
| UCP2 | 11 | -2.47873 | 0.01319 |
| RPUSD4 | 11 | 2.45144 | 0.01423 |
| AASDHPPT | 11 | -2.43469 | 0.0149 |
| IPO7 | 11 | -2.33607 | 0.01949 |
| CCDC88B | 11 | 2.27147 | 0.02312 |
| MED19 | 11 | 2.260993 | 0.02376 |
| TMEM126A | 11 | 2.255799 | 0.02408 |
| TRIM5 | 11 | 2.227169 | 0.02594 |
| TRIM66 | 11 | 2.215632 | 0.02672 |
| EIF3F | 11 | -2.17942 | 0.0293 |
| CRY2 | 11 | -2.1646 | 0.03042 |
| ZNF143 | 11 | -2.14247 | 0.03216 |
| RPS3 | 11 | -2.0933 | 0.03632 |
| BANF1 | 11 | -2.08619 | 0.03696 |
| SNX15 | 11 | 2.067819 | 0.03866 |
| THYN1 | 11 | -2.06382 | 0.03904 |
| CKAP5 | 11 | -2.0199 | 0.04339 |
| C2CD2L | 11 | 2.011244 | 0.0443 |
| NAP1L4 | 11 | -1.99664 | 0.04586 |
| ACER3 | 11 | 1.994067 | 0.04614 |
| MPEG1 | 11 | -1.96233 | 0.04972 |
| TCP11L2 | 12 | -3.24789 | 0.00116 |
| MANSC1 | 12 | 2.45421 | 0.01412 |
| UNC119B | 12 | 2.37141 | 0.01772 |
| WIBG | 12 | 2.36152 | 0.0182 |
| PFKM | 12 | -2.18849 | 0.02863 |
| ACADS | 12 | 2.18695 | 0.02875 |
| SNRNP35 | 12 | -2.18688 | 0.02875 |
| ALDH2 | 12 | 2.08909 | 0.0367 |
| POU6F1 | 12 | -2.04948 | 0.04041 |
| SCYL2 | 12 | 2.00957 | 0.04448 |
| SELPLG | 12 | 1.97889 | 0.04783 |
| CLN5 | 13 | -2.6303 | 0.00853 |
| HSPH1 | 13 | -2.296 | 0.02168 |
| UCHL3 | 13 | -2.2713 | 0.02313 |
| DIS3 | 13 | 2.0404 | 0.04131 |
| USPL1 | 13 | -2.0206 | 0.04332 |
| ZNF410 | 14 | 2.68206 | 0.00732 |
| DHRS7 | 14 | -2.61038 | 0.00904 |
| VRK1 | 14 | -2.49318 | 0.01266 |
| OSGEP | 14 | 2.41595 | 0.01569 |
| SAV1 | 14 | -2.21106 | 0.02703 |
| GPR68 | 14 | -2.17248 | 0.02982 |
| UBR7 | 14 | 2.09906 | 0.03581 |
| GMPR2 | 14 | -2.08645 | 0.03694 |
| IFI27L1 | 14 | 2.03741 | 0.04161 |
| ENTPD5 | 14 | -1.9985 | 0.04566 |
| GPR65 | 14 | -1.97639 | 0.04811 |
| MYO5A | 15 | -3.49331 | 0.000477 |
| ISG20 | 15 | -2.65993 | 0.007816 |
| EHD4 | 15 | 2.56096 | 0.010438 |
| SPTBN5 | 15 | 2.54999 | 0.010773 |
| PLA2G4B | 15 | 2.50891 | 0.01211 |
| SYNM | 15 | 2.45124 | 0.014237 |
| LACTB | 15 | -2.26267 | 0.023656 |
| LEO1 | 15 | 2.2532 | 0.024246 |
| GLCE | 15 | 2.18781 | 0.028684 |
| NIPA1 | 15 | -2.16614 | 0.0303 |
| MAPKBP1 | 15 | 2.15964 | 0.030801 |
| KLF13 | 15 | -2.05925 | 0.03947 |
| CHMP1A | 16 | 3.6124 | 0.000303 |
| HP | 16 | 3.52122 | 0.00043 |
| PRR14 | 16 | -3.01205 | 0.002595 |
| MAPK3 | 16 | -3.00458 | 0.002659 |
| DHX38 | 16 | -2.4407 | 0.014659 |
| PGP | 16 | 2.35103 | 0.018722 |
| SNRNP25 | 16 | -2.21803 | 0.026553 |
| ZFPM1 | 16 | 2.20227 | 0.027646 |
| POLR3K | 16 | 2.10169 | 0.03558 |
| MPHOSPH6 | 16 | -2.09057 | 0.036567 |
| TRAP1 | 16 | -2.07309 | 0.038164 |
| CD2BP2 | 16 | 2.04721 | 0.040637 |
| TCF25 | 16 | 1.99469 | 0.046077 |
| CRLF3 | 17 | -3.27964 | 0.00104 |
| SNX11 | 17 | 3.20209 | 0.00136 |
| SMARCD2 | 17 | -3.03491 | 0.00241 |
| LIMD2 | 17 | 2.80647 | 0.00501 |
| NUP88 | 17 | 2.79169 | 0.00524 |
| CBX4 | 17 | 2.67052 | 0.00757 |
| EVI2B | 17 | 2.6573 | 0.00788 |
| AZI1 | 17 | -2.43055 | 0.01508 |
| CDK5R1 | 17 | -2.38866 | 0.01691 |
| TAX1BP3 | 17 | -2.35033 | 0.01876 |
| CDK5RAP3 | 17 | 2.25203 | 0.02432 |
| SRR | 17 | 2.23837 | 0.0252 |
| RNASEK | 17 | 2.2261 | 0.02601 |
| GALK1 | 17 | 2.17482 | 0.02964 |
| XYLT2 | 17 | 2.16367 | 0.03049 |
| RABEP1 | 17 | -2.16125 | 0.03068 |
| PCGF2 | 17 | -2.1463 | 0.03185 |
| CLEC10A | 17 | 2.05643 | 0.03974 |
| WNT3 | 17 | 2.04165 | 0.04119 |
| RPA1 | 17 | -2.02497 | 0.04287 |
| EVI2A | 17 | -2.00936 | 0.0445 |
| TMEM107 | 17 | 1.99973 | 0.04553 |
| PSMB3 | 17 | 1.98613 | 0.04702 |
| TTC39C | 18 | -2.4566 | 0.014 |
| ANKRD12 | 18 | -2.1784 | 0.0294 |
| CTDP1 | 18 | -2.1042 | 0.0354 |
| VAPA | 18 | -2.0232 | 0.0431 |
| PPP4R1 | 18 | 1.9937 | 0.0462 |
| SERTAD1 | 19 | -3.06446 | 0.00218 |
| SDHAF1 | 19 | -2.99904 | 0.00271 |
| ZNF559 | 19 | 2.73022 | 0.00633 |
| RBM42 | 19 | 2.61377 | 0.00895 |
| DDA1 | 19 | 2.43925 | 0.01472 |
| ARRDC5 | 19 | 2.38852 | 0.01692 |
| RPL36 | 19 | 2.33107 | 0.01975 |
| EPS15L1 | 19 | -2.26984 | 0.02322 |
| VRK3 | 19 | -2.24942 | 0.02449 |
| STK11 | 19 | -2.21273 | 0.02692 |
| EPOR | 19 | 2.18902 | 0.0286 |
| CYTH2 | 19 | -2.15108 | 0.03147 |
| PLAUR | 19 | 2.14851 | 0.03167 |
| SNAPC2 | 19 | 2.09744 | 0.03595 |
| ZSCAN18 | 19 | -2.08659 | 0.03693 |
| ZNF181 | 19 | 2.06477 | 0.03894 |
| OPA3 | 19 | -2.03175 | 0.04218 |
| EVI5L | 19 | -2.0266 | 0.0427 |
| PANK2 | 20 | 2.4866 | 0.0129 |
| ATP5E | 20 | -2.211 | 0.027 |
| TSHZ2 | 20 | -2.1899 | 0.0285 |
| ZNF831 | 20 | 2.0048 | 0.045 |
| MORC3 | 21 | 2.7881 | 0.0053 |
| ITSN1 | 21 | -2.2975 | 0.0216 |
| CBX6 | 22 | -2.40661 | 0.0161 |
| GNB1L | 22 | 2.06241 | 0.0392 |
| CHCHD10 | 22 | -1.9743 | 0.0483 |

Note: TWAS, transcriptome-wide association study; TWAS.Z, TWAS Z-score; TWAS.P, TWAS *P*-value.

**Table S4 List of common genes between TWAS of osteonecrosis and the mRNA expression profile analysis of bone marrow mesenchymal stem cells**

| **Tissues** | **Gene Name** | **Chromosome** | **TWAS.Z** | **TWAS.P** | **Fold Change** |
| --- | --- | --- | --- | --- | --- |
| **Muscle skeleton** | RGPD8 | 2 | 2.7454 | 0.0060 | 2.97 |
|  | ALS2CR12 | 2 | -2.2991 | 0.0215 | 3.00 |
|  | FASTKD1 | 2 | -2.2049 | 0.0275 | 2.06 |
|  | ICA1L | 2 | 2.0797 | 0.0376 | 2.18 |
|  | ANAPC4 | 4 | -2.9989 | 0.0027 | 2.02 |
|  | CCDC125 | 5 | 2.6381 | 0.0083 | 2.32 |
|  | TDRD6 | 6 | 2.6360 | 0.0084 | 13.45 |
|  | SLC25A27 | 6 | -2.0717 | 0.0383 | 3.76 |
|  | GOLM1 | 9 | -2.1483 | 0.0317 | 2.36 |
|  | ITIH5 | 10 | 2.2008 | 0.0278 | 2.67 |
|  | ZNF33B | 10 | -2.1003 | 0.0357 | 2.15 |
|  | KIN | 10 | -1.9915 | 0.0464 | 2.11 |
|  | RABEP1 | 17 | 2.5776 | 0.0099 | 2.22 |
|  | VRK3 | 19 | -2.5178 | 0.0118 | 2.04 |
|  | ZNF100 | 19 | -2.2263 | 0.0260 | 3.38 |
| **Peripheral blood** | MIER1 | 1 | -1.9755 | 0.0482 | 2.34 |
|  | MFSD6 | 2 | 2.0255 | 0.0428 | 2.87 |
|  | PAIP2B | 2 | 1.9624 | 0.0497 | 4.20 |
|  | CCDC125 | 5 | 2.1995 | 0.0279 | 2.32 |
|  | FAM13B | 5 | 2.0959 | 0.0361 | 2.17 |
|  | RSBN1L | 7 | 2.3914 | 0.0168 | 2.15 |
|  | STEAP4 | 7 | 2.2078 | 0.0273 | 7.03 |
|  | GINS4 | 8 | 2.2285 | 0.0259 | 3.04 |
|  | WDR37 | 10 | -2.3495 | 0.0188 | 3.05 |
|  | KDM5A | 12 | -2.3218 | 0.0202 | 2.17 |
|  | TAOK3 | 12 | -2.2725 | 0.0231 | 2.43 |
|  | HP | 16 | 3.4296 | 0.0006 | 2.05 |
|  | CPD | 17 | -2.0947 | 0.0362 | 2.21 |
|  | CEP192 | 18 | 2.3478 | 0.0189 | 2.75 |
|  | LMAN1 | 18 | -2.3066 | 0.0211 | 2.02 |
|  | VRK3 | 19 | -2.3930 | 0.0167 | 2.04 |
|  | KLK1 | 19 | -2.3195 | 0.0204 | 2.12 |
|  | ZNF66 | 19 | -2.0498 | 0.0404 | 2.85 |
| **Whole blood** | USP24 | 1 | 3.0655 | 0.0022 | 2.72 |
|  | JMJD4 | 1 | 2.4444 | 0.0145 | 2.29 |
|  | CEP350 | 1 | 2.1329 | 0.0329 | 2.53 |
|  | DDX1 | 2 | 2.3140 | 0.0207 | 2.05 |
|  | LANCL1 | 2 | -2.2279 | 0.0259 | 3.05 |
|  | MFSD6 | 2 | 2.2229 | 0.0262 | 2.87 |
|  | GCA | 2 | -2.1736 | 0.0297 | 2.01 |
|  | FASTKD1 | 2 | -2.1052 | 0.0353 | 2.06 |
|  | WDR48 | 3 | -2.3258 | 0.0200 | 2.26 |
|  | KLHL24 | 3 | 2.1122 | 0.0347 | 2.36 |
|  | ANAPC4 | 4 | -2.9839 | 0.0029 | 2.02 |
|  | PLA2G12A | 4 | -2.8763 | 0.0040 | 2.06 |
|  | CLK4 | 5 | 2.2277 | 0.0259 | 3.96 |
|  | ENPP4 | 6 | 2.0201 | 0.0434 | 2.44 |
|  | STEAP4 | 7 | 2.1500 | 0.0315 | 7.03 |
|  | RSBN1L | 7 | -1.9900 | 0.0466 | 2.15 |
|  | GKAP1 | 9 | -2.8968 | 0.0038 | 2.01 |
|  | AGTPBP1 | 9 | -2.1507 | 0.0315 | 2.71 |
|  | ERP44 | 9 | -2.0602 | 0.0394 | 3.86 |
|  | ALDH1A1 | 9 | -1.9619 | 0.0498 | 3.16 |
|  | PDLIM1 | 10 | -1.9833 | 0.0473 | 2.14 |
|  | IPO7 | 11 | -2.3361 | 0.0195 | 2.54 |
|  | DIS3 | 13 | 2.0404 | 0.0413 | 2.19 |
|  | USPL1 | 13 | -2.0206 | 0.0433 | 2.44 |
|  | LEO1 | 15 | 2.2532 | 0.0242 | 2.20 |
|  | NIPA1 | 15 | -2.1661 | 0.0303 | 2.56 |
|  | HP | 16 | 3.5212 | 0.0004 | 2.05 |
|  | AZI1 | 17 | -2.4306 | 0.0151 | 2.77 |
|  | RABEP1 | 17 | -2.1613 | 0.0307 | 2.22 |
|  | EVI2A | 17 | -2.0094 | 0.0445 | 4.96 |
|  | PSMB3 | 17 | 1.9861 | 0.0470 | 2.04 |
|  | ANKRD12 | 18 | -2.1784 | 0.0294 | 5.25 |
|  | ARRDC5 | 19 | 2.3885 | 0.0169 | 3.82 |
|  | VRK3 | 19 | -2.2494 | 0.0245 | 2.04 |
|  | ZNF181 | 19 | 2.0648 | 0.0389 | 2.70 |
|  | MORC3 | 21 | 2.7881 | 0.0053 | 2.92 |

Note: TWAS, transcriptome-wide association study; TWAS.Z, TWAS Z-score; TWAS.P, TWAS *P*-value.
